# Supplementary figures and images for: Cellobiose Consumption Uncouples Extracellular Glucose Sensing and Glucose Metabolism in Saccharomyces cerevisiae
Source: mBio. 2017 Aug 8;8(4):e00855-17. doi: 10.1128/mBio.00855-17 (PMC5550752; doi:10.1128/mBio.00855-17)

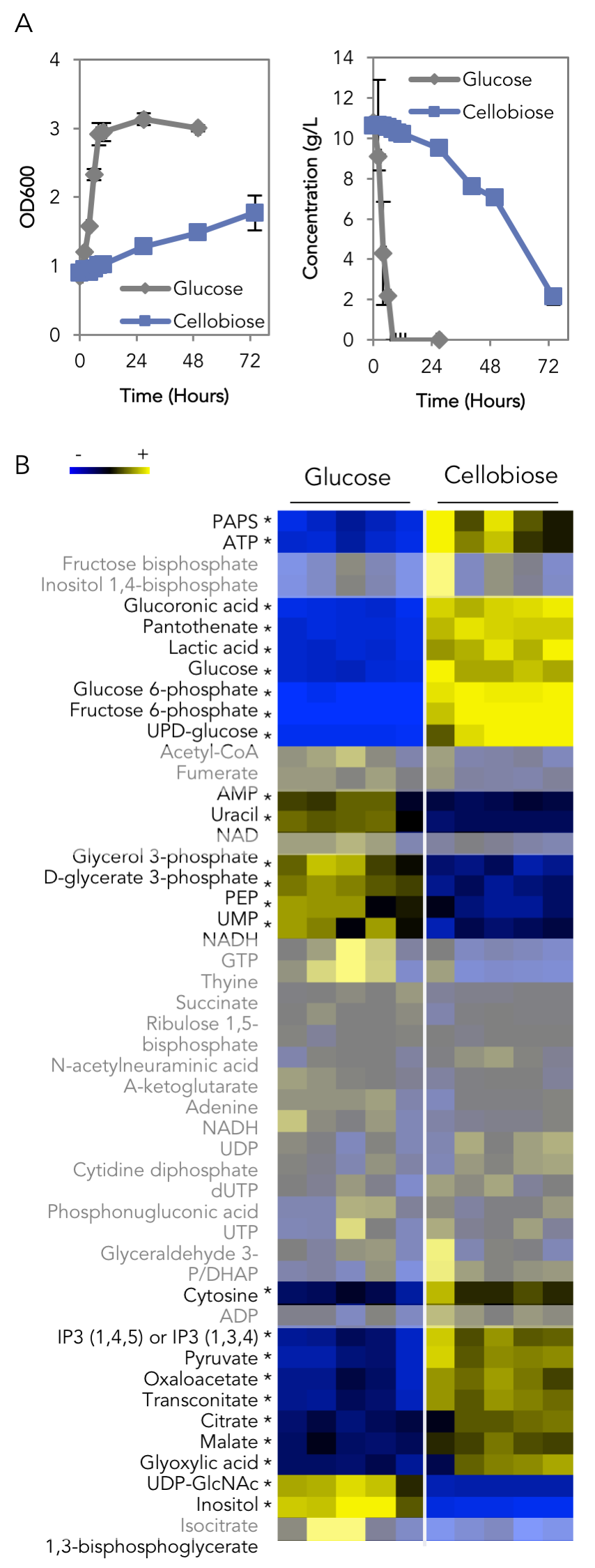

Supplement: FIG S1 [file mbo004173423sf1.tif]

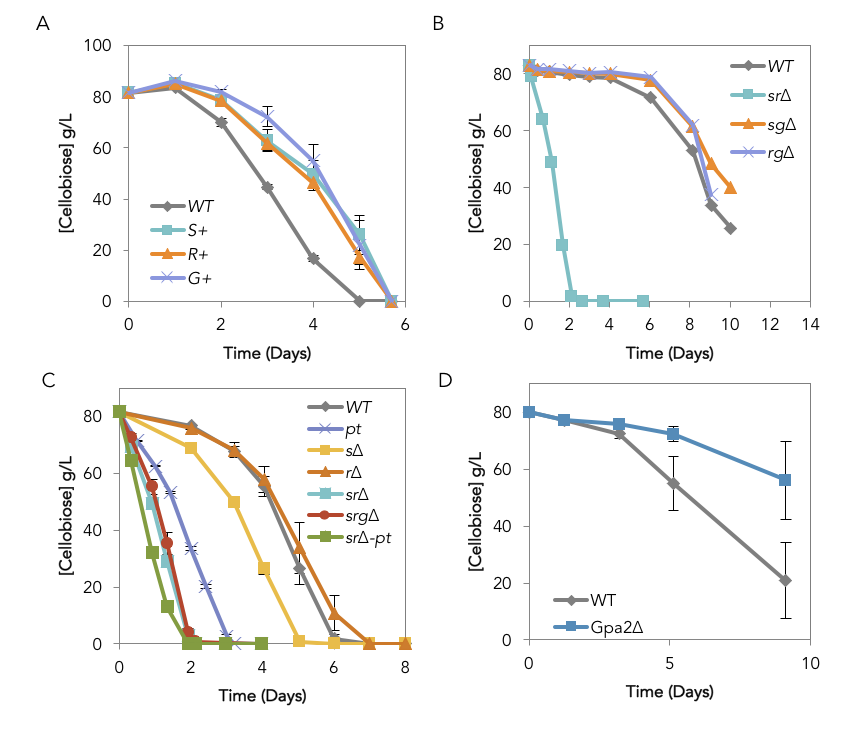

Supplement: FIG S2 [file mbo004173423sf2.tif]

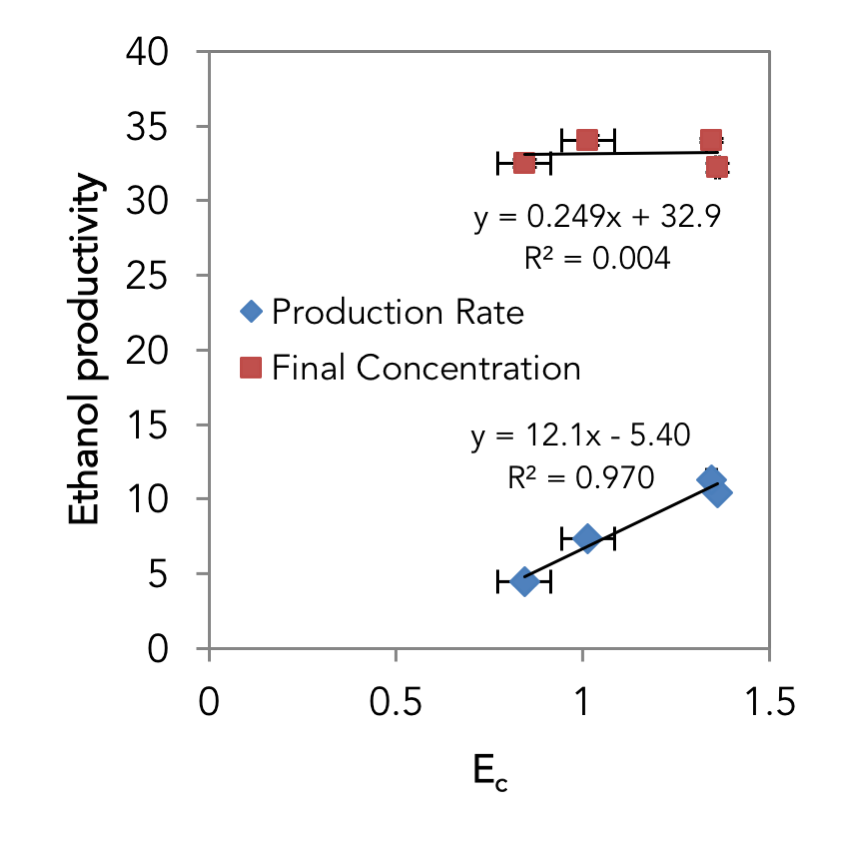

Supplement: FIG S3 [file mbo004173423sf3.tif]
